# FUS contributes to mTOR-dependent inhibition of translation

**DOI:** 10.1074/jbc.RA120.013801

**Published:** 2021-01-13

**Authors:** Myriam Sévigny, Isabelle Bourdeau Julien, Janani Priya Venkatasubramani, Jeremy B. Hui, Paul A. Dutchak, Chantelle F. Sephton

**Affiliations:** Department of Psychiatry and Neuroscience, CERVO Brain Research Centre, Laval University, Quebec City, Quebec, Canada

**Keywords:** mRNA translation, fused in sarcoma (FUS), amyotrophic lateral sclerosis (ALS) (Lou Gehrig disease), frontotemporal degeneration (FTD), neurodegeneration, mTORC1, mTORC2, Torin1, rapamycin, mammalian target of rapamycin (mTOR), translation regulation, RNA-binding protein, ribosome, fragile X mental retardation (FMRP), polyribosome, protein synthesis

## Abstract

The amyotrophic lateral sclerosis (ALS) and frontotemporal dementia (FTD)–linked RNA-binding protein called FUS (fused in sarcoma) has been implicated in several aspects of RNA regulation, including mRNA translation. The mechanism by which FUS affects the translation of polyribosomes has not been established. Here we show that FUS can associate with stalled polyribosomes and that this association is sensitive to mTOR (mammalian target of rapamycin) kinase activity. Specifically, we show that FUS association with polyribosomes is increased by Torin1 treatment or when cells are cultured in nutrient-deficient media, but not when cells are treated with rapamycin, the allosteric inhibitor of mTORC1. Moreover, we report that FUS is necessary for efficient stalling of translation because deficient cells are refractory to the inhibition of mTOR-dependent signaling by Torin1. We also show that ALS-linked FUS mutants R521G and P525L associate abundantly with polyribosomes and decrease global protein synthesis. Importantly, the inhibitory effect on translation by FUS is impaired by mutations that reduce its RNA-binding affinity. These findings demonstrate that FUS is an important RNA-binding protein that mediates translational repression through mTOR-dependent signaling and that ALS-linked FUS mutants can cause a toxic gain of function in the cytoplasm by repressing the translation of mRNA at polyribosomes.

The amyotrophic lateral sclerosis (ALS) and frontotemporal dementia (FTD) are fatal neurodegenerative diseases that share overlapping clinical and pathological features (1, 2, 3). ALS is marked by the progressive degeneration of upper and lower motor neurons, which lead to loss of motor function and paralysis. FTD is characterized by the degeneration of neurons in the frontal and temporal lobes, which negatively impacts cognition, language, and behavior. It is estimated that up to 40–50% of ALS patients have clinical features of FTD with the occurrence of frontotemporal atrophy, and ∼50% of FTD cases present with subclinical motor neuron degeneration (1, 2, 3). The majority of ALS and FTD cases are sporadic; however, 5–10% of familial ALS cases are associated with autosomal dominant mutations in the gene encoding *fused in sarcoma* (*FUS*), which correspond with its nuclear depletion and cytoplasmic aggregation in neurons and glia (4, 5, 6). Although *FUS* mutations are rarely found in FTD patients (7, 8), cytoplasmic pathological aggregates of WT FUS are observed in subgroups of FTD (9, 10). Together, the pathological and genetic findings for ALS and FTD indicate that a cytoplasmic “toxic gain of function” may underlie a common mechanism for these diseases.

FUS, also called TLS (translocated in sarcoma), is a ubiquitously expressed RNA-binding protein involved in diverse cellular functions (11). It is a 526-amino acid protein that is predominantly localized to the nucleus where it regulates transcription, splicing, and DNA damage repair (12, 13, 14, 15, 16, 17). The protein contains a C-terminal, nonclassical proline-tyrosine nuclear localization sequence and a central nuclear export sequence that regulates nuclear-cytoplasmic shuttling (18, 19, 20). In the cytoplasm, FUS has numerous roles in RNA metabolism, including transport and stability, microRNA processing, and translation regulation (21, 22, 23, 24, 25). Studies have shown that FUS binds to several thousand RNAs at coding, noncoding, and 5´- and 3´-UTR regions (12, 13, 14, 15), mediated through its RNA recognition motif, zinc finger domain, and three arginine-glycine-glycine (RGG) boxes (26, 27, 28, 29). FUS also contains an N-terminal low-complexity domain that has been shown to affect its liquid-phase properties, as well as its interactions with RNA and other proteins (30, 31, 32).

Recent studies have suggested that FUS is involved in the regulation of protein synthesis. ALS-linked *FUS* mutations in the proline-tyrosine nuclear localization sequence domain localize predominantly to the cytoplasm, which correlate with a reduction in protein synthesis (20, 33, 34). Changes in mRNA trafficking and stability (23, 24, 35, 36), as well as aberrant protein–protein interactions (37, 38, 39), have all been attributed to these pathological *FUS* mutations. Although WT FUS is mainly localized in the nucleus, its localization to the cytoplasm can also be enhanced in response to conditions that restrict translation. In neurons, glutamate excitotoxicity has been shown to induce the cytoplasmic localization of FUS bound to glutamate ionotropic receptor AMPA type subunit 2 (GRIA2) mRNA, which corresponds to reduced global protein synthesis (36). In other conditions where protein synthesis is repressed, such as heat shock, sodium arsenite, or sorbitol treatments, FUS is found in cytoplasmic stress granules composed of messenger ribonucleoproteins and stalled mRNAs (40, 41, 42, 43). The cytoplasmic localization of FUS has been shown to be regulated by post-translational modifications at phosphorylation sites by Src kinase and at methylation sites by *N*-arginine methyltransferase 1 (PRMT1), which influence its interaction with transportin (TNPO1) and promote FUS nuclear import (18, 44, 45, 46). Modifying the phosphorylation or methylation status of ALS-linked FUS mutants can promote its redistribution back to the nucleus and reverse its cytotoxic effects (34, 44, 45, 47).

The mTOR signaling pathway integrates both intra- and extracellular stimuli responsible for regulating metabolism, protein synthesis, and cellular growth (48, 49). Two structurally distinct multiprotein complexes called mTOR complex 1 (mTORC1) and mTOR complex 2 (mTORC2) both contain the mTOR kinase subunit that targets diverse substrates for phosphorylation (48, 49). mTORC1 functions as a growth factor, nutrient, and energy sensor that controls protein synthesis through the phosphorylation of downstream targets like S6K1/2 (p70 ribosomal S6 protein kinases 1 and 2) (48, 49, 50, 51). Subsequently, S6K1/2 phosphorylates and activates substrates that promote mRNA translation, including the ribosomal protein S6 (52, 53). mTORC2 is activated by growth factor signaling and controls cytoskeletal dynamics by phosphorylating members of the AGC family of protein kinases, including AKT (54, 55). Although mTORC1 and mTORC2 are activated through defined signal transduction cascades, the cellular processes regulated by these kinase complexes are not easily distinguishable because of the cross-talk between the pathways. Alterations in the mTOR signaling pathway have been observed in ALS and FTD patients and in models of disease (56, 57, 58).

Our study investigates the functional role of cytoplasmic FUS in regulating protein synthesis through its association with polyribosomes. Here we show that FUS associates with stalled polyribosomes and that this association is enhanced in response to mTOR inhibition. Intriguingly, we show that pharmacological inhibition of the mTOR kinase with Torin1 but not the allosteric inhibitor of mTORC1, rapamycin, increases FUS association with polyribosomes. These data suggest that FUS activity on polyribosomes is modulated by mTORC2-dependent signal transduction. We also show that FUS-deficient cells are refractory to translational inhibition by Torin1 treatment. Next, we examined the association of ALS-linked FUS mutants, R521G and P525L, with polyribosomes and found that these mutants associate more abundantly with polyribosome fractions and reduce translation. Remarkably, point mutations in the RGG2 RNA-binding domain of FUS reduces its RNA-binding affinity (29) and decreases the dominant-negative effect of ALS-linked FUS mutants. Together, these data suggest that FUS can negatively regulate translation through its association with polyribosomes in an RNA-binding dependent manner and that its activity on polyribosomes is dynamically regulated through mTOR activity.

## Results

### FUS associates with stalled polyribosomes in an mTOR-dependent manner

**Figure 1:**
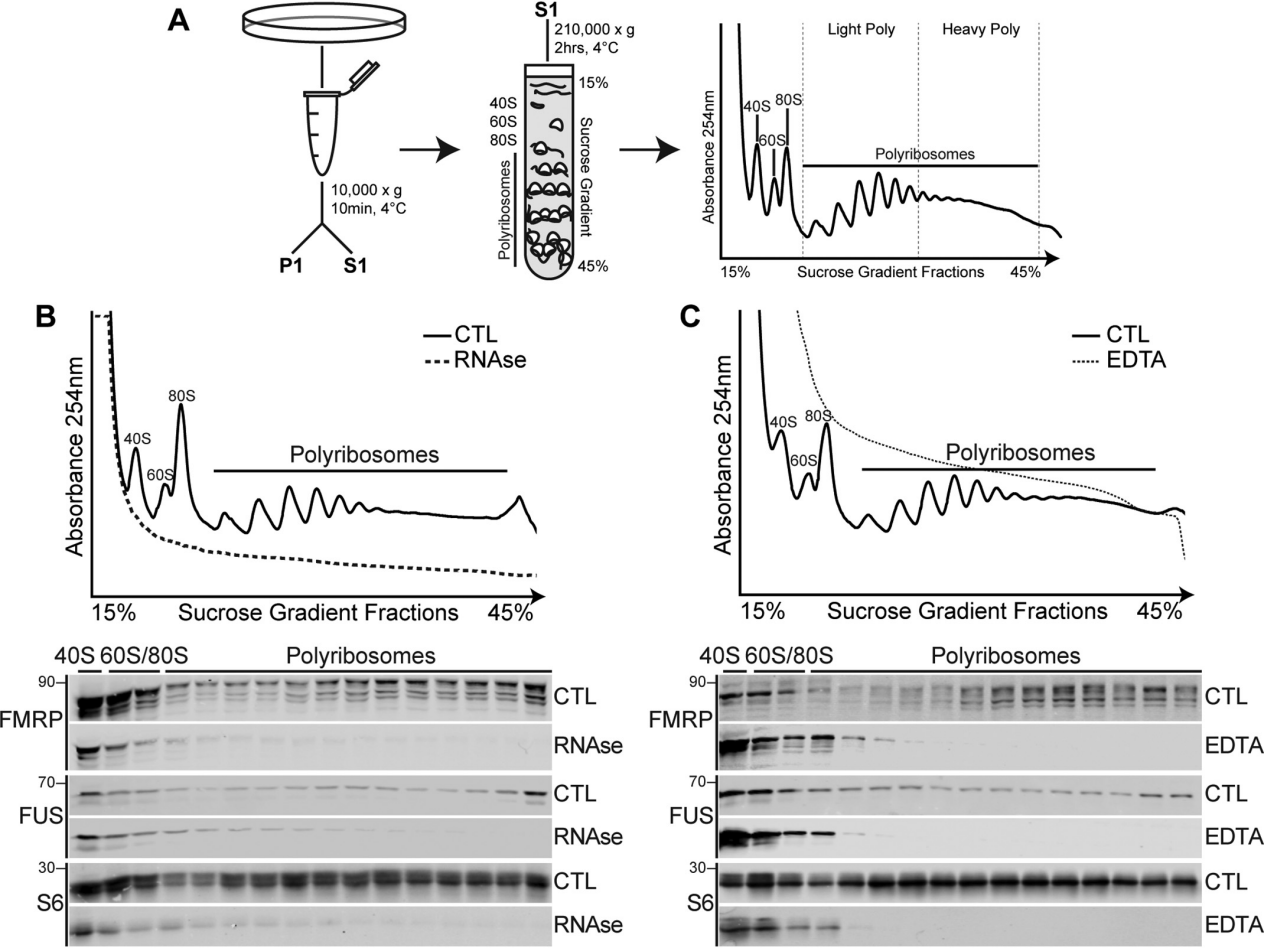
**FUS associates with polyribosomes in an RNA- and protein-dependent manner.***A*, schematic of polyribosome isolation by sucrose gradient fractionation. *S1*, soluble fraction; *P1*, pellet. Indicated on the absorbance traces are ribosomal subunits 40S and 60S, monosomes (*80S*), light polyribosomes (*Light Poly*), and heavy polyribosomes (*Heavy Poly*). *B* and *C*, S1 extracts obtained from HEK293T cells were treated with RNase A (*B*, 400 µg/ml, 10 min, 37 °C) or EDTA (*C*, 30 mm, 20 min, on ice), and polyribosomes were fractioned. Absorbance (254 nm) traces of total RNA distribution (*top panels*) and Western blots of proteins isolated from S1 sucrose gradient fractions (*bottom panels*) blotted with antibodies against FUS, FMRP, and the ribosomal protein S6, a marker for the 40S subunit. The data shown are representative of *n* = 3 biological replicates. *CTL*, control.

To determine the role of FUS in regulating translation, we biochemically purified polyribosomes from HEK293T cells using sucrose gradient fractionation methods (Fig. 1*A*). We found that FUS is present in the 40S, 60S, and monosome fractions, as well as light and heavy polyribosome fractions (Fig. 1*B*). We then characterized the co-sedimentation behavior of FUS with polyribosomes by treating cell extracts with RNase A, to degrade RNA, or EDTA, to chelate Mg^2+^ and dissociate protein complexes (59, 60). We found that both treatments disrupt the sedimentation behavior of FUS, as indicated by its shift toward low-density, free ribosomal fractions (Fig. 1, *B* and *C*). We also examined FMRP (fragile X mental retardation 1), a RNA-binding protein that has been shown to interact with FUS (61) and whose association with polyribosomes has been well-characterized (62, 63, 64). Consistent with previous reports, we found that FMRP is reduced in light and heavy polyribosomes by these treatments, similar to FUS. Collectively, these data indicate that the presence of FUS in polyribosome fractions depends on RNA and protein interactions.

**Figure 2:**
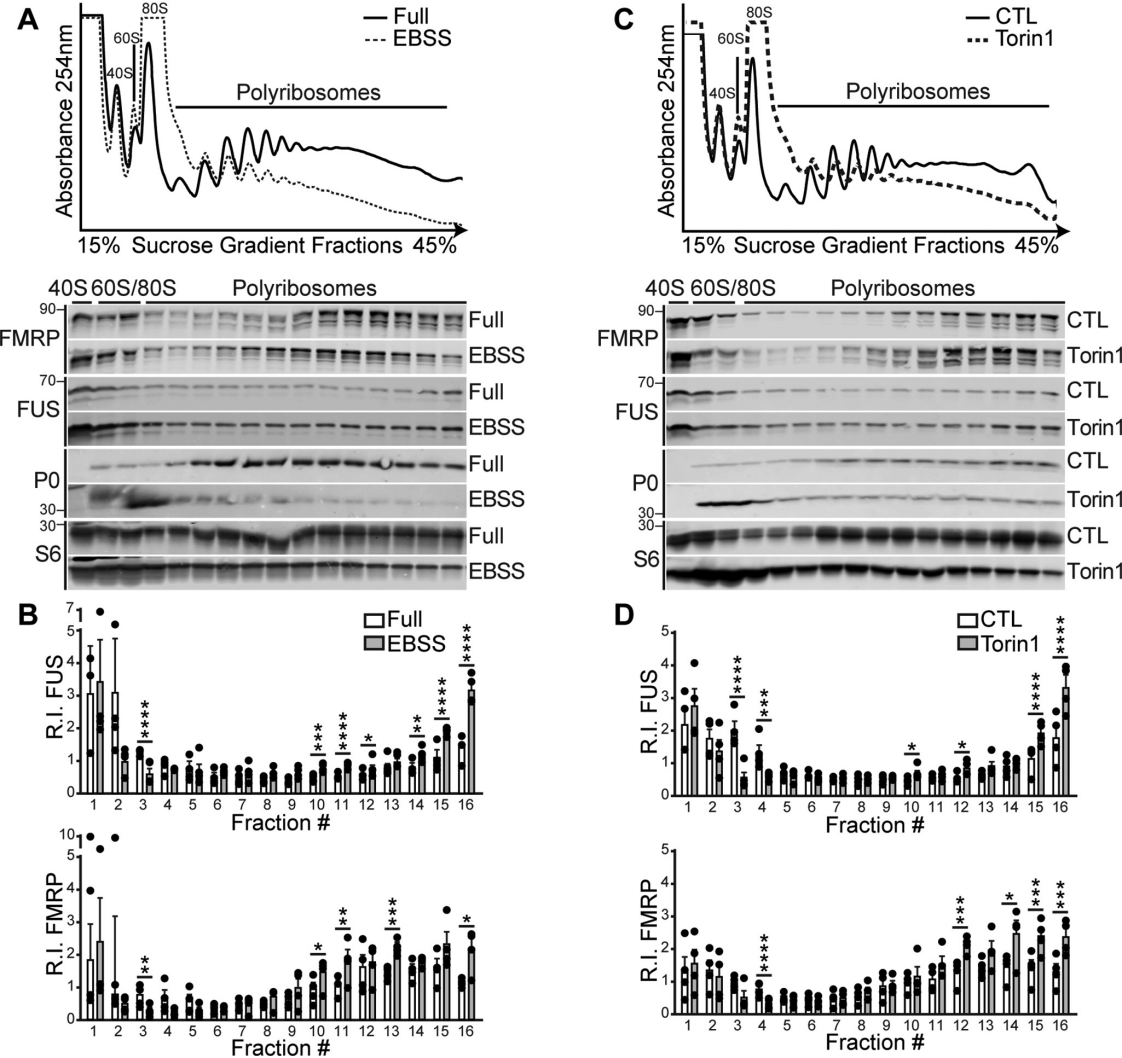
**FUS associates with polyribosomes in an mTOR-dependent manner.***A* and *C*, HEK293T cells were cultured in complete media (*A*, *Full*) or starved in EBSS (2 h) or treated with DMSO (control, *CTL*) or the mTOR kinase inhibitor Torin1 (*C*, 250 nm, 2 h). Absorbance (254 nm) traces of total RNA distribution (*top panels*) and Western blots of proteins isolated from S1 sucrose gradient fractions (*bottom panels*) blotted with antibodies against FUS, FMRP, and ribosomal proteins S6 and P0. *B* and *D*, quantification of the protein R.I. from each fraction relative to control. Statistical analysis was performed using a repeated measures ANOVA for *n* = 4 biological replicates. *, *p* < 0.05; **, *p* < 0.01; ***, *p* < 0.005; ****, *p* < 0.001. The *error bars* represent ± S.E.

**Figure 3:**
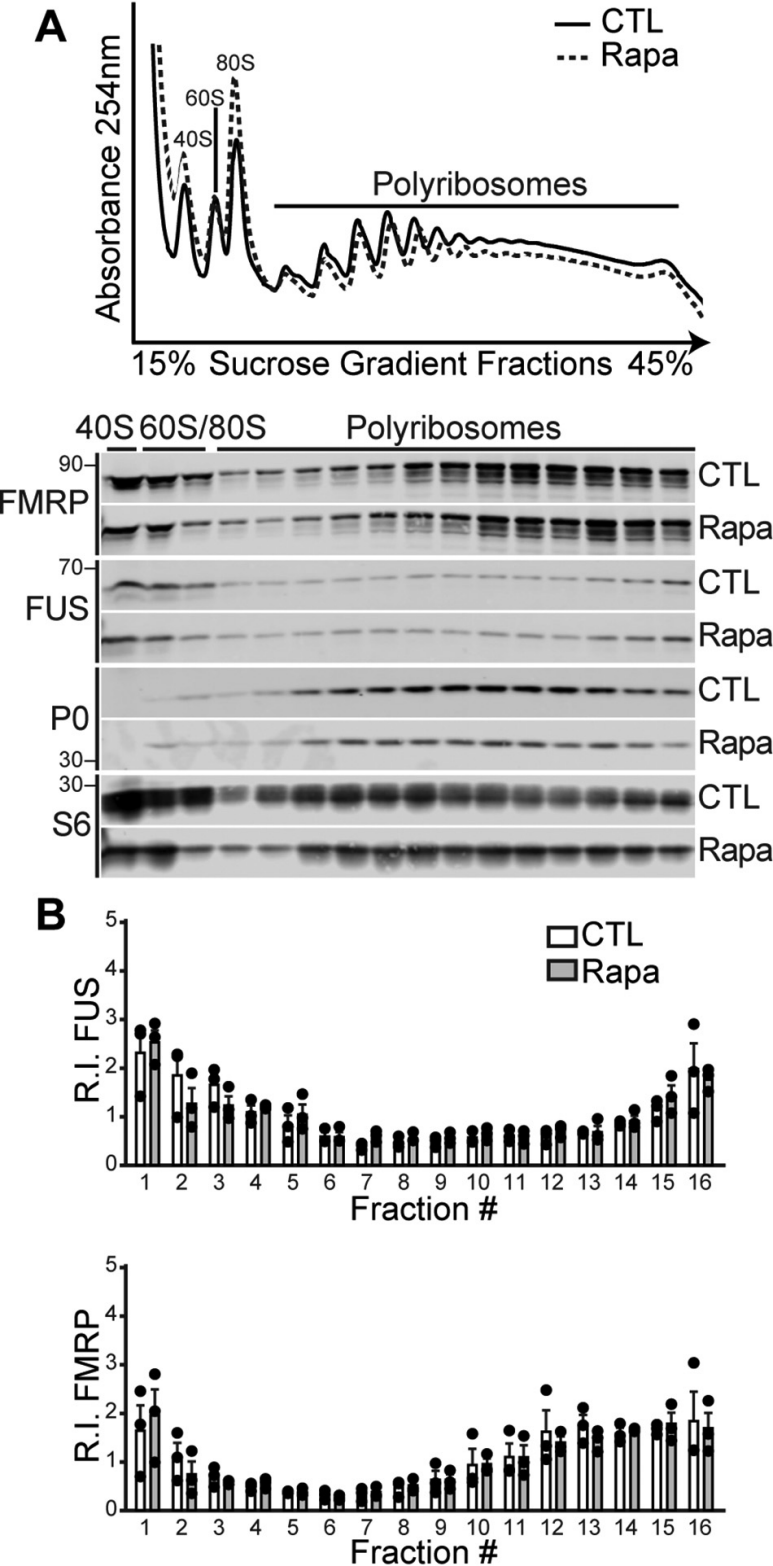
**FUS does not associate with polyribosomes in response to mTORC1 inhibition.***A*, HEK293T cells were treated with DMSO (control, *CTL*) or the mTORC1 inhibitor rapamycin (*Rapa*, 10 nm, 2 h). Absorbance (254 nm) trace of total RNA distribution (*top panel*) and Western blots of proteins isolated from S1 sucrose gradient fractions (*bottom panel*) blotted with antibodies against FUS, FMRP, and ribosomal proteins S6 and P0. *B*, quantification of the protein R.I. from each fraction relative to control. Statistical analysis was performed using a repeated measures ANOVA for *n* = 3 biological replicates. The *error bars* represent ± S.E.

Given that FUS has been implicated as a negative regulator of translation (20, 23, 24, 33, 34, 35), we investigated the association of FUS with polyribosomes in conditions where translation is impaired. First, we cultured cells in Earle's balanced salt solution (EBSS), an amino acid–deficient medium that impairs translation and reduces mTOR signaling (65, 66). We observed a significant increase in FUS with polyribosome fractions (Fig. 2, *A* and *B*). Next we treated cells with Torin1, a pharmacological inhibitor of the mTOR kinase subunit that is required for mTORC1 and mTORC2 function. We observed a significant increase in FUS with heavy polyribosomes (Fig. 2, *C* and *D*), similar to what we observed following EBSS treatment. We also observed FUS in monosome fractions treated with Torin1, although at lower levels than heavy polyribosomes (Fig. 2, *C* and *D*), suggesting that FUS may also impact translation at monosomes (67, 68). Under these treatment conditions, we also determined the association of FMRP with polyribosomes and observed that it responds in a similar manner as FUS when treated with EBSS or Torin1 (Fig. 2). In contrast, when cells were treated with the allosteric inhibitor of mTORC1, rapamycin, we did not observe a change in the association of FUS or FMRP with polyribosomes (Fig. 3). Collectively, our data suggest that FUS and FMRP interactions with polyribosomes depend on mTORC2 kinase activity.

**Figure 4:**
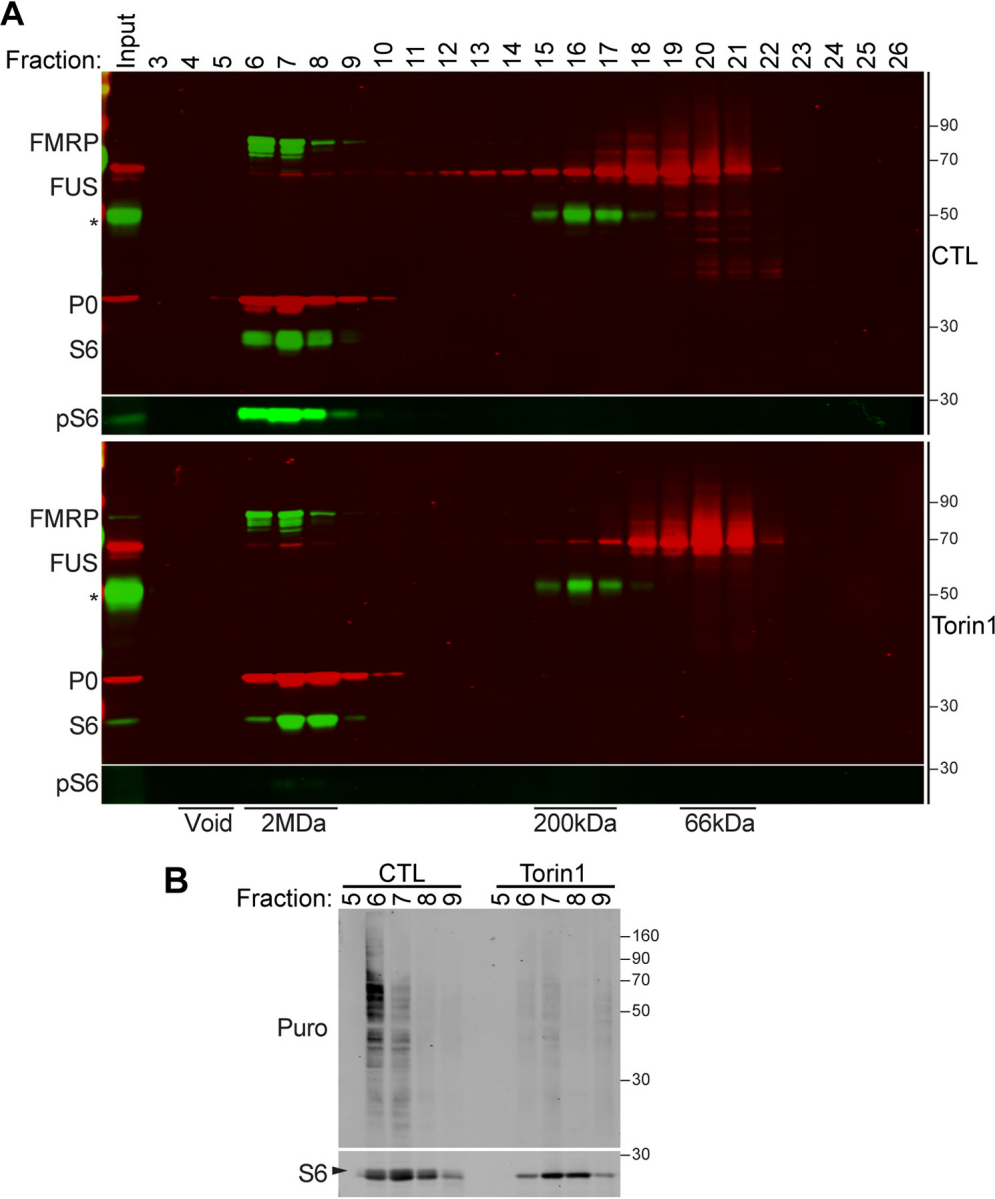
**FUS and FMRP associate with large molecular mass complexes.** HEK293T cells were treated with DMSO (control, *CTL*) or Torin1 (250 nm, 2 h), and S1 fractions were separated by mass using a Superdex 200 10/300 GL size-exclusion column. *A*, Western blots of fractioned proteins blotted with antibodies against FUS, FMRP, total S6, and phospho-S6 (*pS6*) and P0. An *asterisk* represents a nonspecific immunoreactive band. *B*, Western blotting of *in vitro* puromycylation of SEC fractions 5–9 isolated from HEK293T cells treated with DMSO (control, *CTL*) or Torin1 and blotted for anti-puromycin (*Puro*) and anti-S6. An *arrow* points to the upper S6 band, which corresponds with the pS6 band detected in *A*. The data shown are representative of *n* = 2 biological replicates.

To further characterize the association of FUS with polyribosomes, we performed size-exclusion FPLC and Western blotting analysis to examine large molecular mass complexes. As shown in Fig. 4*A*, FUS and FMRP are present in a ∼2-MDa complex with S6 (40S marker) and P0 (60S marker), corresponding with the mass of polyribosomes (69). When cells were treated with Torin1, we observed that FUS and FMRP are maintained in the ∼2-MDa complex, along with dephosphorylated S6 (Fig. 4*A*). These data are consistent with FUS and FMRP association with stalled polyribosomes. We performed *in vitro* puromycin-labeling assays to assess the synthesis of nascent polypeptide chains in fractions 5–9 and found a reduction in protein synthesis in fractions obtained from Torin1-treated cells compared with control (Fig. 4*B*). Because previous reports have shown that FUS localization to the cytoplasm can be enhanced in response to translation repression (36, 42, 70), we examined the cytoplasmic distribution of FUS in response to Torin1. Using subcellular fractionation of control or Torin1-treated cells, we observed a modest but statistically significant increase in FUS in the cytoplasmic fraction (Fig. S1). Together, these observations further support a role for FUS in translation regulation at polyribosomes in response to mTOR inhibition.

**Figure 5:**
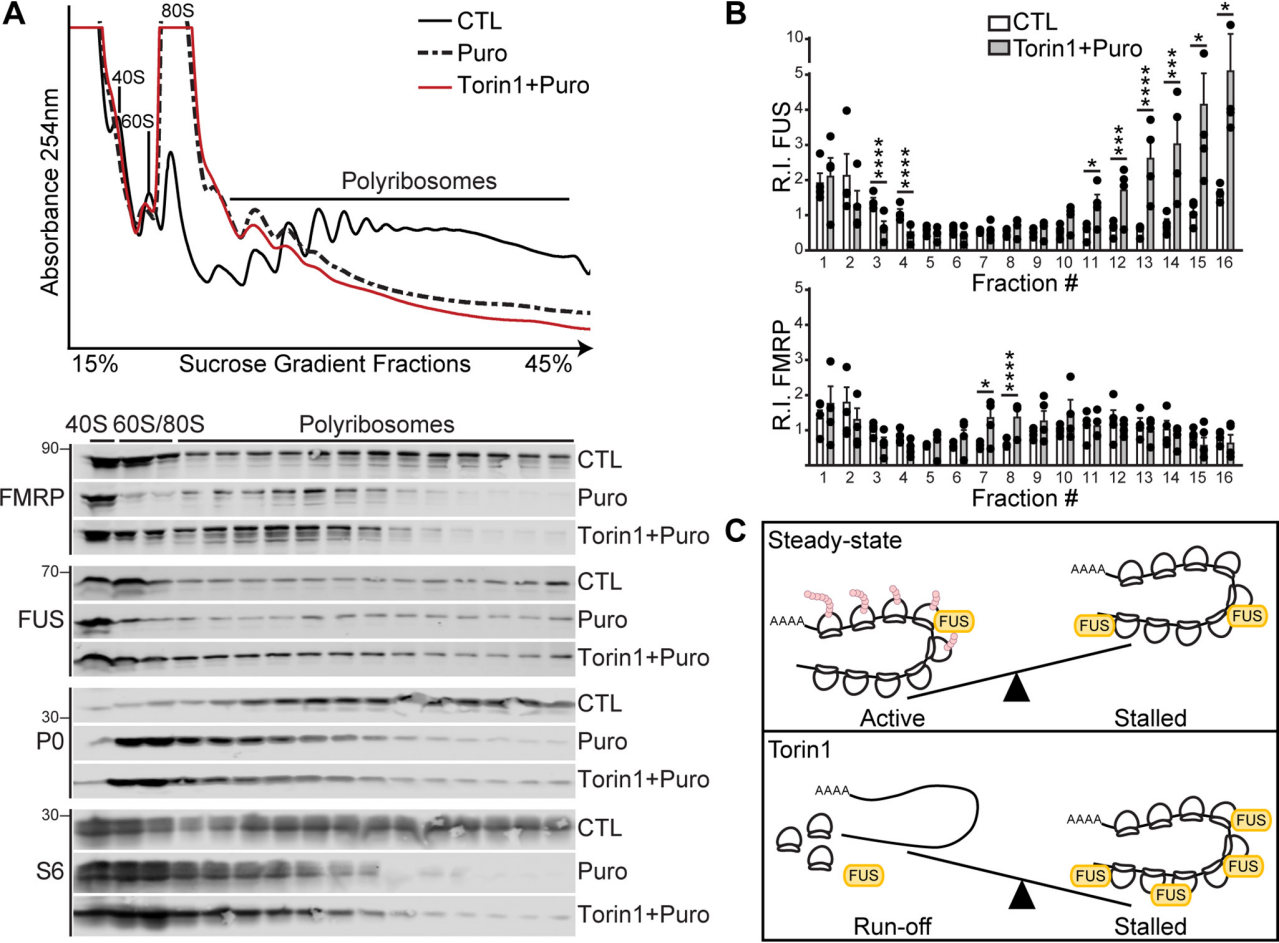
**FUS associates with stalled polyribosomes in an mTOR-dependent manner.***A*, HEK293T cells were treated with puromycin (*Puro*, 1 mm, 1 h) to induce active ribosome dissociation from mRNA or Torin1 (250 nm, 2 h) with puromycin (1 mm, 1 h) dosed into the media for the final hour of treatment (Torin1 + Puro) or DMSO (control, *CTL*). Absorbance (254 nm) trace of total RNA distribution (*top panel*) and Western blots of proteins isolated from S1 sucrose gradient fractions (*bottom panel*) blotted with antibodies against FUS, FMRP, and ribosomal proteins S6 and P0. *B*, quantification of the protein R.I. from each fraction obtained from Torin1+Puro-treated cells relative to control. Statistical analysis was performed using a repeated measures ANOVA for *n* = 4 biological replicates. *, *p* < 0.05; ***, *p* < 0.005; ****, *p* < 0.001. The *error bars* represent ± S.E. *C*, *top panel*, under steady-state conditions, FUS associates with active and stalled polyribosomes. *Bottom panel*, in response to Torin1 treatment, FUS that is associated with active polyribosomes runs off, concurrent with a more abundant association of FUS with stalled polyribosomes.

We then investigated whether FUS associates with active or stalled polyribosomes. We treated cells with puromycin, a tRNA analog that becomes incorporated into the nascent polypeptide chain causing premature termination and dissociation of active ribosomes from mRNA. Following puromycin treatment, we observed a shift of S6 and P0 ribosomal markers toward the light polyribosome fractions, along with FMRP and FUS (Fig. 5*A*). Remarkably, we also observed that some FUS remained in heavy polyribosome fractions, consistent with stalled polyribosomes (Fig. 5*A*). To test whether the increase in FUS association with polyribosomes in response to Torin1 occurs at stalled polyribosomes, we pretreated cells with Torin1 and added puromycin to induce active ribosome release from mRNA. We observed more FUS associated with polyribosomes when compared with cells treated with puromycin or vehicle alone (Fig. 5). Because Torin1 blocks translation initiation and causes runoff of active ribosomes from mRNA (71), these data suggest that FUS localizes to stalled polyribosomes when mTOR kinase is inhibited. These results are consistent with our previous observations that show FUS is more abundant on polyribosomes when mTOR is repressed (Fig. 2, *C* and *D*). Interestingly, under these treatment conditions, we observed that FMRP is more abundant in the lighter polyribosome fractions (Fig. 5, *A* and *B*), suggesting that the mechanism of translation regulation by FMRP and FUS in response to mTOR inhibition is distinct. Together, these data show that FUS can associate with stalled polyribosomes and that this interaction is regulated in an mTOR-dependent manner.

### FUS contributes to mTOR-dependent translational repression

**Figure 6:**
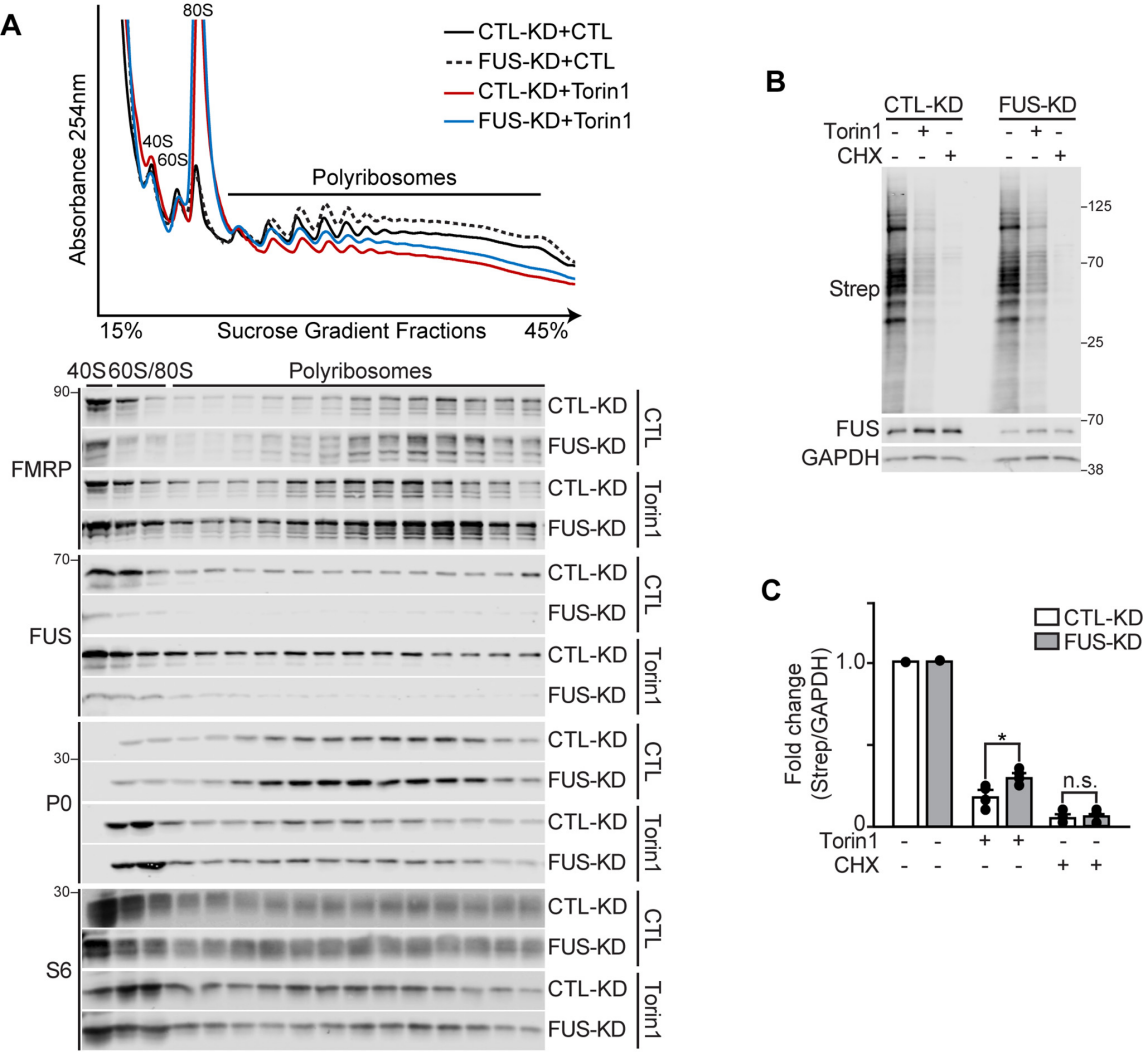
**FUS-depleted cells have reduced sensitivity to translation inhibition by Torin1.** HEK293T cells were infected with shRNAs against a nontargeted shRNA (CTL-KD) or FUS (FUS-KD) and treated with DMSO (control, *CTL*) or Torin1 (250 nm, 2 h). *A*, Western blots of proteins isolated from S1 sucrose gradient fractions blotted with antibodies against FUS, FMRP, and ribosomal proteins S6 and P0. The data shown are representative of *n* = 3 biological replicates. *B*, HEK293T cells infected with CTL-KD or FUS-KD were metabolically labeled with Click-iT® AHA to assess nascent protein synthesis. Proteins were processed for Western blotting and detected using antibodies against FUS, GAPDH and streptavidin (*Strep*), which detects AHA-labeled nascent proteins. *C*, quantification of AHA-labeled nascent proteins (*Strep*) relative to CTL-KD (DMSO). The proteins were normalized to GAPDH. Statistical analysis of *C* was performed using a Student's *t* test from *n* = 4 biological replicates. *n.s.*, nonsignificant *p* > 0.05; *, *p* < 0.05. The *error bars* represent ± S.E.

We investigated the contribution of FUS in mediating translation repression when mTOR is inhibited by Torin1. We performed these experiments by infecting HEK293T cells with lentivirus containing nontargeting shRNAs (CTL-KD) or FUS shRNAs (FUS-KD) and compared the effects of Torin1 on polyribosomes in these conditions. In response to Torin1, the absorbance profiles for polyribosome fractions from FUS-deficient cells showed slightly more absorbance in the polyribosome fractions, compared with CTL-KD–treated cells (Fig. 6*A*). When we examined the sedimentation pattern of the ribosomal subunit markers P0 and S6 by Western blotting, we observed a trend toward a higher abundance of ribosomal proteins in the polyribosome fractions of FUS-KD cells treated with Torin1, compared with CTL-KD–treated cells (Fig. S2, A and C). Interestingly, we also observed more FMRP on heavy polyribosomes in FUS-KD cells treated with Torin1, compared with CTL-KD–treated cells (Fig. 6*A* and Fig. S2, B and C).

Previous studies show that deletion of eukaryotic translation initiation factor 4E-binding proteins (4E-BP1/2) can render cells resistant to translation inhibition by Torin1 (71). We investigated whether FUS could also function in a similar manner. We treated CTL-KD and FUS-KD cells with DMSO (CTL) or Torin1 and labeled nascent proteins with l-azidohomoalanine (AHA). We did not observe a change in global protein synthesis in FUS-KD cells when compared with CTL-KD cells in vehicle-treated conditions (Fig. 6*B*). In contrast, when we treated cells with Torin1, we found that depleting cells of FUS rendered them partially insensitive to translation inhibition (Fig. 6, *B* and *C*, and Fig. S2D). To investigate the effect of FUS on translational signaling pathways, we examined the phosphorylation status of downstream targets of mTOR kinase, including S6K, 4E-BP1, and AKT. We found that knocking down FUS did not impact these pathways as determined by Western blotting analysis (Figs. S3 and S4). Taken together, our data show that depleting cells of FUS renders cells less sensitive to Torin1-dependent inhibition of translation, supporting a novel mechanism of FUS-dependent translational stalling. These data are consistent with our observations that FUS contributes to translational stalling in response to mTOR inhibition.

### ALS–FUS mutants repress translation at polyribosomes in an RNA binding–dependent manner

**Figure 7:**
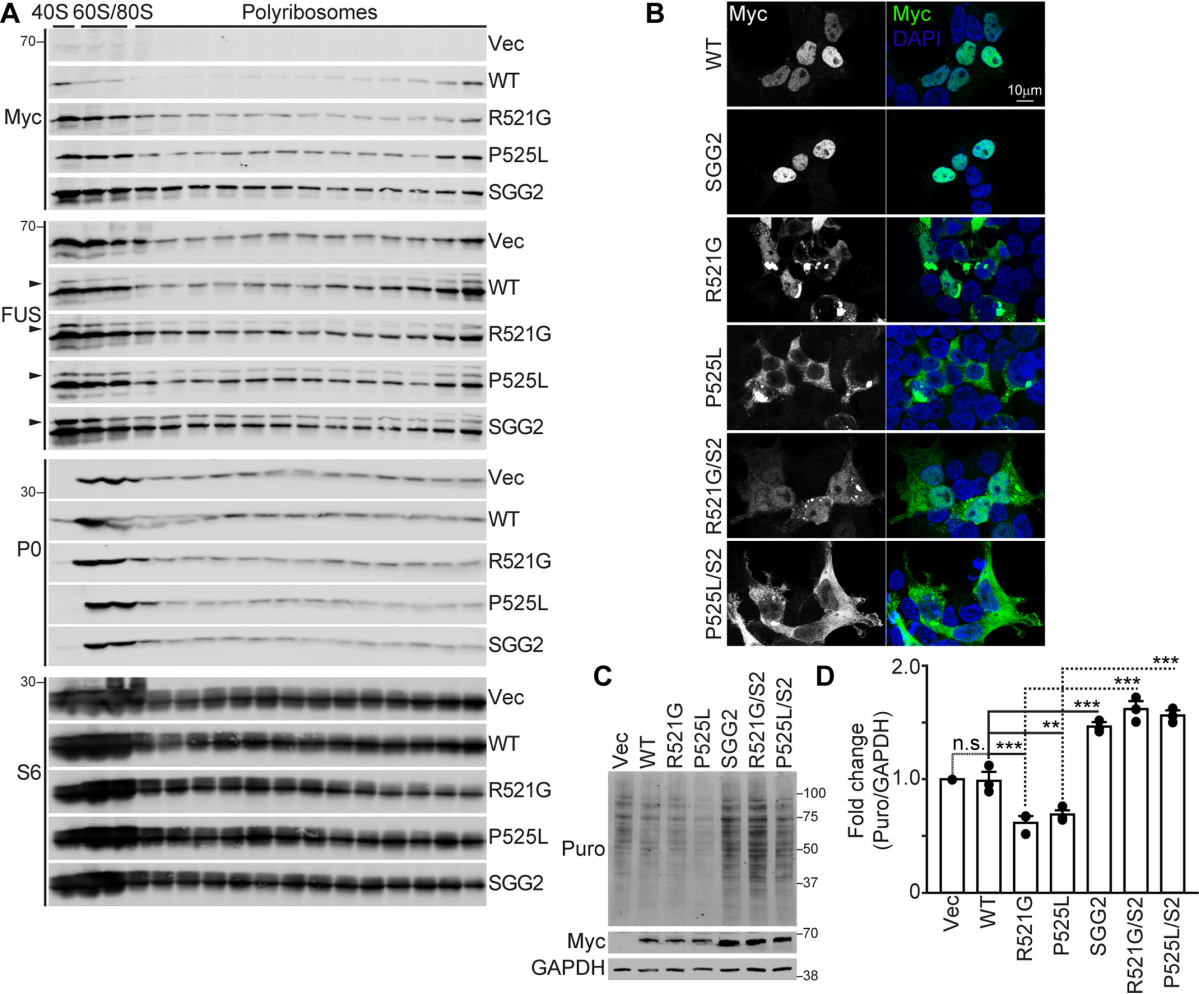
**ALS-FUS mutants repress translation in an RNA-dependent manner.***A*, HEK293T cells were transfected with empty vector pcDNA4b (*Vec*) or Myc-tagged FUS constructs: WT FUS (*WT*), ALS-FUS mutants (R521G and P525L), and FUS (SGG2), which has reduced binding affinity for RNA (29), for 48 h before S1 extracts were subjected to polyribosome isolation by sucrose gradient centrifugation followed by Western blotting. An *arrow* points to exogenous expression of Myc-tagged FUS detected by anti-FUS. The data shown are representative of *n* = 3 biological replicates. *B*, confocal microscopy images of HEK293T cells transfected with Myc-tagged WT, R521G, P525L, SGG2 mutant, or ALS-FUS and SGG2 combination mutations R521G/S2 and P525L/S2. Shown are antibodies against Myc to label exogenous FUS (*green*) and 4′,6-diamino-2-phenylindole (*DAPI*, *blue*) to label the nucleus. *C*, Western blots of puromycin-labeled (*Puro*, 1 µg/ml, 20 min) nascent proteins from HEK293T cells transfected with the indicated vectors blotted with antibodies against puromycin, Myc, and GAPDH. *D*, quantification of puromycin-labeled nascent proteins relative to Vec. The proteins were normalized to GAPDH. Statistical analysis of *D* was performed using a Student's *t* test from *n* = 3 biological replicates. *n.s.*, nonsignificant *p* > 0.05; **, *p* < 0.01; ***, *p* < 0.005. The *error bars* represent ± S.E.

We then asked whether ALS-linked autosomal dominant mutations in *FUS*, which localize predominately to the cytoplasm (20, 33, 34, 35), could affect translation by interacting with polyribosomes. We overexpressed ALS-linked FUS R521G and P525L mutants in cells and found that these proteins did not have a major effect on polyribosome abundance, compared with empty vector (Fig. S5). However, when we examined the association of ALS-FUS mutants with polyribosomes, we found that both mutants were more abundant in the 40S, 60S, and monosome fractions, as well as polyribosome fractions, when compared with FUS-WT (Fig. 7*A*). We then investigated whether the interaction of FUS with polyribosomes depends on its ability to bind RNA. We generated the SGG2 mutations in FUS that have been shown to reduce its RNA-binding affinity (29). We found that the SGG2 mutant was enriched in the 40S, 60S, monosome, and polyribosome fractions, when compared with FUS-WT (Fig. 7*A*), but we did not observe any changes to the nuclear localization of this mutant using immunocytochemistry (Fig. 7*B*). These findings show that the ability of FUS to bind RNA affects its association with polyribosomes.

**Figure 8:**
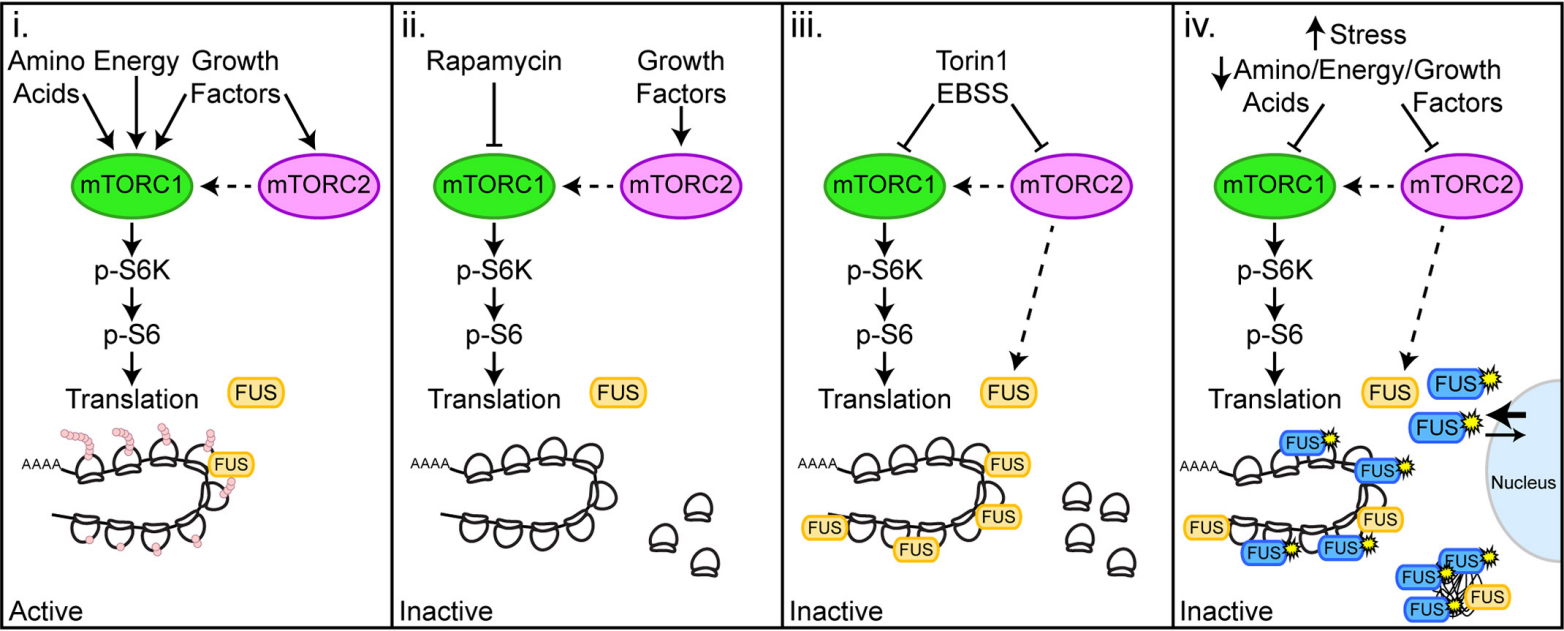
**Model of FUS-dependent mRNA translation regulation.***Panel i*, activation of the mTORC1 and mTORC2 pathways promotes monosome and polyribosome assembly onto mRNA for active translation. *Panel ii*, inhibition of mTORC1 by rapamycin causes translational arrest, without affecting FUS association with polyribosomes. *Panel iii*, inhibition of mTORC1 and mTORC2 by EBSS (amino acid depletion) and Torin1 result in defects in translation initiation, which lead to active polyribosome runoff and translation inhibition. When mTORC2 is inhibited, FUS is recruited to polyribosomes to promote translation inhibition and polyribosome stalling. *Panel iv*, ALS-FUS R521G and P525L mutants that localize more prominently to the cytoplasm also associate more abundantly with polyribosomes to inhibit translation and protein synthesis.

Next, we examined the impact of ALS-FUS R521G and P525L mutants on nascent protein synthesis in cells using puromycin labeling of nascent proteins. We observed a repression of global protein synthesis in cells expressing FUS R521G and P525L (Fig. 7, *C* and *D*), consistent with previous reports (20, 23, 24, 33, 34, 35). In contrast, cells expressing the FUS SGG2 mutant showed an increase in global protein synthesis (Fig. 7, *C* and *D*). To test the contribution of RNA binding on the repression of protein synthesis by the ALS-FUS mutants, we generated the SGG2 mutations in ALS-FUS R521G and P525L expression vectors. Remarkably, R521G/S2 and P525L/S2 double-mutants showed more global protein expression when compared with the R521G and P525L mutants alone (Fig. 7, *C* and *D*). We examined the steady-state localization of the R521G/S2 and P525L/S2 mutants using immunocytochemistry and found that SGG2 mutations do not impact the cytoplasmic localization caused by the ALS-FUS mutations (Fig. 7*B*). These findings suggest that FUS regulates translation through its association with polyribosomes and RNA. These data support our model that cytoplasmic FUS, which is prevalent in ALS and FTD, contributes to translational stalling of polyribosomes through RNA binding–dependent interactions (Fig. 8).

## Discussion

Our studies have revealed a novel function of FUS in repressing translation through its association with polyribosomes. Previous studies have suggested that FUS can negatively impact protein synthesis (12, 13, 14, 15) and that changes in its subcellular localization, as with ALS-linked FUS mutants, correlate with altered rates of protein synthesis (20, 23, 24, 33, 34, 35). We now show that FUS associates with stalled polyribosomes and that this interaction is enhanced by the inhibition of mTORC2 (Figure 2, Figure 3, Figure 4, Figure 5, Figure 6). Consistent with previous claims of a toxic gain of function in the cytoplasm of ALS-linked FUS mutants, our data support the idea that cytoplasmic retention of FUS increases its proximity to polyribosomes for stalling to occur (Fig. 7). Cellular changes that compromise mTORC2 signal transduction, including reduced growth factor signaling, subsequently limit the translation of polyribosomes with FUS recruitment. Our data suggest that ALS-linked FUS mutants that are found predominantly in the cytoplasm could sensitize patients to translational deficiencies that occur with decreased growth factor signaling through mTORC2, leading to the rapid progression of ALS.

Our results are consistent with FUS functioning as a negative regulator of translation (20, 23, 24, 33, 34, 35). First, we have demonstrated that FUS associates with monosomes and polyribosomes using gradient centrifugation and size-exclusion chromatography methods (Figure 1, Figure 2, Figure 3, Figure 4). These results are consistent with previous proteomic screens that have observed FUS interactions with ribosomal subunits (24). Second, the association of FUS with polyribosomes is enriched in response to Torin1, a pharmacological inhibitor of the mTOR kinase (Figure 2, Figure 3, Figure 4, Figure 5). Intriguingly, our pharmacological studies show that inhibition of mTORC2, not mTORC1, increases the interaction of FUS with stalled polyribosomes (Figure 2, Figure 3). These data are consistent with a coordination of FUS and ribosomal activities that can occur through mTORC2 and mTORC1 pathways, respectively. Third, depleting FUS from cells renders them refractory to the repressive effect on translation by Torin1, which supports the role of FUS as an inhibitor of translation (Fig. 6 and Fig. S2D). Future studies are needed to determine whether the targets of FUS-stalled polyribosomes are identical to transcripts that are exported from the nucleus by FUS.

The functional implications for FUS in the cytoplasm are underscored by our observations that ALS-FUS mutants are enriched in polyribosome fractions and repress global protein synthesis (Fig. 7*A*, *C* and *D*). Importantly, when mutations are made in the RGG2 region of the ALS-FUS mutants, which reduces their binding affinity to RNA, we demonstrate a significant rescue in global protein synthesis (Fig. 7, *C* and *D*). These observations show that FUS can repress translation at polyribosomes and that its binding to RNA is required for efficient repression of translation. Although various protein–protein interactions with FUS have been identified through proteomic analysis (24), our data suggest that targeting the FUS RGG2 domain represents a potential means to prevent the toxic effect of ALS-FUS mutants.

While investigating the dynamic interaction of FUS with polyribosomes, we uncovered that FUS activity is modulated through the mTORC2 signaling pathway (Figure 2, Figure 3). Indeed, previous studies show that FUS cytoplasmic localization and mRNA trafficking activity can be modulated through intracellular signaling pathways. Activation of mGluR1/5 (metabotropic glutamate receptors 1 and 5) with agonists results in FUS localization and mRNA trafficking to distal dendrites where protein synthesis is controlled (19, 20, 22). In response to glutamate excitotoxicity, FUS localizes to the cytoplasm and may repress protein synthesis of target mRNAs (36). Other cellular stressors like heat shock or sodium arsenite cause FUS to form cytoplasmic stress granules (40, 41, 42, 43), which form transiently to stall RNA translation (43, 72).

Here we show an increase of FUS on polyribosomes in response to Torin1 treatment (Fig. 2, *C* and *D*), concomitant with an increase of FUS in the cytoplasm (Fig. S1), in contrast to the mTORC1 inhibitor, rapamycin (Fig. 3). Indeed, these findings are consistent with FUS acting as a repressor of translation on polyribosomes through impaired mTORC2 kinase activity (Fig. 8). We also observed that FUS remains present on monosomes in cells treated with Torin1, although less abundant than on heavy polyribosomes (Fig. 2, *C* and *D*), suggesting that FUS may also impact translation at monosomes (67, 68). Furthermore, we demonstrate that FUS-KD cells are refractory toward Torin1 (Fig. 6 and Fig. S2D). Although translation was reduced in FUS-KD by Torin1, it was not inhibited to the extent of control cells. These data are consistent with Torin1 inhibiting both mTORC1 and mTORC2, which are known to impact distinct steps of translation. We also observe a trend toward a greater abundance of ribosomal subunits in the polyribosome fractions in the FUS-KD cells treated with Torin1, compared with CTL-KD cells (Fig. S2A). These findings suggest that FUS may regulate a subset of mRNAs under conditions where mTOR is inhibited, such that in the absence of FUS, certain transcripts are translated. These data indicate that other mechanisms of translational stalling are not sufficient to compensate for the loss of FUS in response to Torin1 treatment (Fig. 6 and Fig. S2). Together, these findings provide evidence that signaling pathways act in different ways to direct FUS activity, determining its subcellular localization, its interactions with proteins and mRNAs, and its role in translation.

To our knowledge, no previous studies directly link FUS activity to mTOR signaling. However, there is evidence to support that FUS is regulated downstream of this pathway. For instance, mTORC2 is a regulator of cytoskeleton rearrangement (73), and FUS has been shown to regulate the translation of cytoskeletal mRNAs (22, 35) and effect dendritic branching and spine formation (19, 20). Additionally Src kinase has found to be a regulator of amino acid–mediated activation of mTORC1 (74), it is also the upstream kinase of FUS, shown to promote nuclear localization of FUS through phosphorylation (46). More work will be required to determine how mTOR signaling coordinates FUS regulation of protein synthesis because of the cross-talk between the mTORC1 and mTORC2 signaling pathways (48, 49, 50).

Our study shows that ALS-linked FUS R521G and P525L mutants, which localize predominantly to the cytoplasm, are enriched on polyribosomes (Fig. 7). Importantly, expression of ALS-FUS mutants significantly inhibit translation compared with FUSWT (Fig. 7, *C* and *D*). We anticipate that this effect occurs because there is more cytoplasmic FUS, which increases the probability for interaction with binding sites in the polyribosome (Fig. 7). Remarkably, the inhibitory effect of ALS-FUS mutants on translation are lost by reducing its affinity to bind RNA by introducing SGG2 mutations in the RGG2 domain (Fig. 7*C* and *D*) (29). Notably, when the ability of FUS to interact with RNA is impaired by these mutations, we observed more FUS in polyribosome fractions corresponding with an increase in nascent protein synthesis (Fig. 7). In our study, we did not examine other biochemical properties of SGG2 mutations in FUS. However, recent studies have shown that the RGG2 domain is regulated by post-translation modifications (44, 45) and contribute to the liquid-phase properties of FUS (75). These properties of FUS could further impact its association with RNA and polyribosomes and its ability to stall translation. Based on our study, therapeutic approaches that target mTOR inhibition for the treatment of ALS (56, 57, 76) should be cautioned in cases of ALS patients with *FUS* mutations.

Previous studies have shown FMRP can directly interact with FUS (61), share mRNA targets (77), and colocalize with FUS in RNA granules (40, 61). In our study, we show that FMRP is enriched on stalled polyribosomes in response to mTOR inhibition with Torin1, like FUS (Figure 2, Figure 3, Figure 4). However, their distribution within the stalled polyribosome fractions was distinct (Fig. 5*A*), suggesting that the way these proteins exert their effect on translation does not occur in the same manner. In FUS-deficient cells treated with Torin1, we observe that FMRP is enriched in heavy polyribosome fractions (Fig. 6*A* and Fig. S2, B and C), suggesting that there may be some aspect of compensation by FMRP to repress translation in the absence of FUS. The activity of FMRP has previously been linked with mTOR signaling, in which mGluR-dependent activation of mTOR/S6K1 caused the phosphorylation of FMRP and its rapid degradation (78), which promoted ribosome re-entry into active translation (79). Recently, FMRP has been shown to regulate 5´-terminal oligopyrimidine tracts motif–containing mRNAs (80), a subset of mRNAs that are translated in response to activation of mTOR. Although FMRP activity has not been previously linked with mTORC2, similar to FUS, FMRP is shown to regulate the translation of mRNAs that are involved in cytoskeletal remodeling and proper dendritic branching and spine formation (81). Together, findings from our study provide new evidence to support the conclusion that FUS and FMRP are involved in repressing translation at polyribosomes in an mTOR-dependent manner.

In summary, we describe a new mechanism by which FUS regulates translation. We conclude that the activity of FUS on polyribosomes can be regulated through the mTORC2 signaling pathway, and under these conditions it can act as a repressor of translation (Fig. 8). Moreover, our findings provide new evidence that ALS-linked FUS mutants promote cytoplasmic toxicity at polyribosomes (Fig. 8). These studies have defined a new biological function of FUS as an important regulator of translation in cells in response to the mTORC2 signal transduction pathway.

## Experimental procedures

### Material

Torin1 (catalog no. 10997) and rapamycin (catalog no. 13346) were from Cayman Chemical, and puromycin (catalog no. 4089) was from Tocris. PhosSTOP (catalog no. 4906845001), cOmplete EDTA-free protease inhibitor mixture (catalog no. hRNAs: TRCN0000001132 (pLKO.1-puro shFUS, FUS-KD1), TRCN0000001133 (pLKO.1-puro shFUS, FUS-KD2), and SHC002 (pLKO.1-puro nonmammalian shRNA control, CTL-KD) were from Sigma–Aldrich. RNase A (catalog no. EN0531), cycloheximide (CHX) (catalog no. AC357420010), goat anti-rabbit Alexa Fluor® 488 antibody (catalog no. 11836170001), MISSION® sA-11034), Click-iT^TM^ protein reaction buffer kit (catalog no. C10276), Click-IT^TM^ AHA (l-azidohomoalanine) (catalog no. C10102), and biotin alkyne (PEG4 carboxamide–propargyl biotin) (catalog no. B10185) were from Thermo Fisher Scientific. DC^TM^ protein assay kit II (catalog no. 5000112) was from Bio-Rad. PDL-coated coverslips are from Neuvitro Corporation (catalog no. GG-12-PDL). Primary antibodies and their sources are listed in Table 1. IRDye® 800CW streptavidin (catalog no. 926-32230) and secondary antibodies IRDye® 680RD goat anti-mouse IgG (catalog no. 926-68070), IRDye® 800CW goat anti-rabbit IgG (catalog no. 926-32211), and IRDye® 680RD goat anti-rabbit IgG (catalog no. 925-68071) are from LI-COR Biosciences.

**Table 1:** Primary antibodies used for Western blot and immunofluorescence analysis

Antibody	Species^a^	Company	Dilution^b^
4E-BP1	R	Cell Signaling Technology 9452 (Whitby, Ontario, Canada)	WB: 1/2000
Akt (pan)	M	Cell Signaling Technology 2920 (clone 40D4 Whitby, Ontario, Canada)	WB: 1/1000
FMRP	R	Abcam ab17722 (Toronto, Ontario, Canada)	WB: 1 /5000
FUS/TLS	R	Proteintech Group 11570-1-AP (Rosemont, IL, USA)	WB: 1 /2000
FUS/TLS	M	Santa Cruz sc-47711 (clone 4H11, Dallas, TX, USA)	WB: 1/2000; IF: 1/500
GAPDH	R	Millipore Sigma G9545 (Oakville, Ontario, Canada)	WB: 1/50,000
Lamin A/C	M	Cell Signaling Technology 4777 (clone 4C11, Whitby, Ontario, Canada)	WB: 1/2000
Myc	R	Abcam ab9106 (Toronto, Ontario, Canada)	WB: 1/5000; IF: 1/2500
p70 S6 kinase	R	Cell Signaling Technology 9202 (Whitby, Ontario, Canada)	WB: 1/2000
Phospho-4E-BP1 (Thr^37/46^)	R	Cell Signaling Technology 2855 (clone 236B4 Whitby, Ontario, Canada)	WB: 1/2000
Phospho-Akt (Ser^473^)	R	Cell Signaling Technology 4060 (clone D9E Whitby, Ontario, Canada)	WB: 1/2000
Phospho-p70 S6 kinase (Thr^389^)	R	Cell Signaling Technology 9205 (Whitby, Ontario, Canada)	WB: 1/1000
Phospho-S6 (Ser^240/244^)	R	Cell Signaling Technology 2215 (Whitby, Ontario, Canada)	WB: 1/2000
Puromycin	M	Millipore Sigma MABE343 (Oakville, Ontario, Canada)	WB: 1/5000
RPLP0	M	Santa Cruz sc-293260 (clone 1B4, Dallas, TX, USA)	WB: 1/5000
S6	R	Cell Signaling Technology 2217 (clone 5G10, Whitby, Ontario, Canada)	WB: 1/10000
S6	M	Cell Signaling Technology 2317 (clone 54D2, Whitby, Ontario, Canada)	WB: 1/1000


a R, rabbit host; M, mouse host.

b IF, immunofluorescence; WB, Western blotting.

### Cell culture and lentivirus production

HEK293T cells were cultured in complete medium (10% fetal bovine serum (Gibco, catalog no. 12483020) and Dulbecco's modified Eagle's medium high-glucose medium (Gibco, catalog no. 11965-092)) and grown under standard culture conditions (37 °C, 5% CO_2_, 95% air). For lentivirus production, HEK293T cells were grown to 60–70% confluence, followed by co-transfection with lentivirus packaging vectors (VSVG and Δ8.9) and a pLKO.1-puro vectors (CTL-KD, FUS-KD1 and FUS-KD2) using FuGENE 6 (Promega, catalog no. E2691) following the manufacturer's instructions. 48 h post-transfection, the condition medium was filtered through a 0.45-μm filter, snap-frozen in liquid nitrogen, and stored at −80 °C until use.

### Western blotting

Proteins were prepared in 1× Laemmli buffer and boiled (5 min, 95 °C) before being resolved on SDS-polyacrylamide gels and transferred to nitrocellulose membranes. The membranes were blocked with 5% nonfat dried skim milk in TBS containing 0.1% (w/v) Tween 20 (TBST) for 1 h at room temperature and incubated with primary antibodies (Table 1) overnight at 4 °C. After washing three times for 10 min with TBST, the membranes were incubated with species-appropriate fluorescent LI-COR secondary antibodies for 1 h at room temperature, washed three times for 10 min with TBST, and imaged using the LI-COR Odyssey imaging system. Analysis of signal intensity was done using Image Studio Lite software, version 5.2.

### Polyribosome fractionation and purification

HEK293T cells were pretreated with 100 μg/ml CHX for 5 min, washed once with ice-cold 1× PBS, pH 7.4 (Gibco, catalog no. 10010-023), containing 100 μg/ml CHX and lysed in polyribosome lysis buffer (PLB) (20 mm Tris-HCl, pH 7.4, 5 mm MgCl_2_, 100 mm KCl, 1% Nonidet P-40, 1 mm DTT, 20 units/μl SUPERase inhibitor, 1× protease inhibitors EDTA-free, 1× PhosSTOP, and 100 μg/ml CHX). The lysates were centrifuged (10,000 × *g*, 10 min, 4 °C) to obtain soluble (S1) and pellet (P1) fractions. Protein determination was performed on S1 fractions using the DC^TM^ protein assay kit II, and equal protein amounts were loaded onto a continuous sucrose gradient (15–45% (w/w) sucrose, 20 mm Tris-HCl, pH 7.4, 100 mm KCl, 5 mm MgCl_2_) and centrifuged in a SW-41Ti rotor (210,000 × *g*, 2 h, 4 °C). The sucrose gradient was fractionated using a BR-188 density gradient fractionation system (Brandel) into 18 fractions (600 μl/fraction) using a sensitivity setting of 1, a baseline setting of 20, and a flow rate of 1.5 ml/min. Throughout the collection, the fractions were monitored by UV absorbance (254 nm). Each fraction was then precipitated in a 3:1 volume of ethanol and incubated overnight at −20 °C. The precipitants were pelleted by centrifugation (16,000 × *g*, 20 min, 4 °C), resuspended in 1× Laemmli buffer, and boiled (5 min, 95 °C). RNase A (400 μg/ml, 10 min, 37 °C) and EDTA (30 mm, 20 min, 4 °C) treatments were performed on the S1 fractions prior to sucrose gradient fractionation. Torin1 (250 nm, 2 h), rapamycin (10 nm, 2 h), and puromycin (1 mm, 1 h) were added directly to the cell culture medium prior to lysis in PLB. The protein relative intensity (R.I.) for each fraction was calculated based the absolute signal intensity for each protein and expressed as a percentage of the total signal. For the statistical analysis using repeated measures ANOVA, the R.I. values obtained from biological replicates were standardized to S6, as the internal control.

### Size-exclusion chromatography

Size-exclusion chromatography (SEC) was performed as previously described (82). S1 fractions were filtered through a 0.45-μm filter, and 12 mg of protein was loaded onto a Superdex 200 10/300 GL column. The samples were eluted using 20 mm Tris-HCl, pH 7.4, 5 mm MgCl_2_, and 1 mm KCl buffer at a flow rate of 0.5 ml/min. A total of 48 fractions were collected at 0.5 ml/fraction, and proteins from fractions 3–26 were processed for Western blotting. The *in vitro* puromycylation labeling was performed by adding 2 μg/ml puromycin and 1× protease inhibitors EDTA-free to SEC fractions, followed by incubation for 15 min at 37 °C. The proteins were precipitated by 20% (w/v) TCA and processed for Western blotting. Molecular mass calibration was carried out by using a gel filtration molecular weight markers kit (Sigma–Aldrich, catalog no. MW-GF-1000).

### Puromycin labeling of nascent proteins

HEK293T cells were transfected using FuGENE 6 according to the manufacturer's instructions. 48 h post-transfection, the cells were harvested for total cell lysates in radioimmune precipitation assay lysis buffer (20 mm Tris-HCl, pH 8.0, 1 mm EDTA pH 8.0, 0.5 mm EGTA, pH 8.0, 1% Triton X-100, 150 mm NaCl, 1 mm DTT, 1× protease inhibitors EDTA-free, and 1× PhosSTOP) and precleared by centrifugation (18,200 × *g*, 30 min, 4 °C) or treated with 1 μg/ml puromycin (20 min, 37 °C) to label nascent proteins. Puromycin-treated cells were washed once with ice-cold 1× PBS, pH 7.4, and lysed in PLB. The samples were precleared by centrifugation (10,000 × *g*, 10 min, 4 °C) to obtain the S1 fraction and processed for Western blotting. Puromycin-labeled nascent proteins were detected using primary antibodies against puromycin.

### AHA labeling

HEK293T cells were infected for 3 days with CTL-KD, FUS-KD1, or FUS-KD2 under puromycin (1 μg/ml) selection before being treated with DMSO, Torin1 (250 nm, 2 h), or CHX (100 μg/ml, 90 min). In the last 1 h of treatments, Click-iT® AHA (50 μm) was added to the cell culture medium. The cells were harvested in PLB and incubated (30 min, 4 °C) in the Click-iT® reaction mixture containing 20 μm biotin-alkyne following the manufacturer's instructions. The proteins were then processed for Western blotting, and IRDye® 800CW Streptavidin was used to detect AHA-labeled nascent proteins.

### Immunofluorescence

Immunofluorescence of HEK293T cells were performed as previously described (83). HEK293T cells were grown on PDL-coated coverslips and fixed with 4% paraformaldehyde for 20 min at room temperature. The samples were washed three times for 5 min in 1× PBS + 0.1 m glycine and then incubated in blocking/permeabilization solution (1× PBS, pH 7.4, 0.2% Triton X-100, 10% goat serum, 0.1% NaAz) for 30 min at room temperature. The samples were incubated in primary antibodies at 4 °C overnight. The samples were washed three times for 10 min with 1× PBS and incubated in Alexa Fluor® 488 secondary antibodies diluted in secondary solution (1× PBS, pH 7.4, 0.1% Triton X-100, 1% goat serum, 0.1% NaAz) for 1 h at room temperature. The coverslips were washed three times for 10 min with 1× PBS and then mounted with ProLong^TM^ Gold antifade mounting media containing 4′,6-diamino-2-phenylindole (Thermo Fisher, catalog no. P36935). Cell images were acquired with a ZEISS LSM 700 confocal microscope using a 63× oil objective and imaged as Z-stacks (1.0-μm step size). Maximum intensity projections were generated, and the images were processed in Fiji ImageJ.

### Site-directed mutagenesis

Human FUS cDNA was cloned into pcDNA4b and then used as a template to generate the pcDNA4b-FUS-SGG2 mutant by site-directed mutagenesis. Oligonucleotides used for mutagenesis are described in Table 2.

**Table 2:** Oligonucleotides used for site-directed mutagenesis

Mutation	Primer	Sequence
R394S	Forward	5´-AGGACCCATGGGCAGTGGAGGCTATGG-3´
	Reverse	5´-CCATAGCCTCCACTGCCCATGGGTCCT-3´
R377S	Forward	5´-TCGCCGGGCAGACTTTAATAGCGGTGGTGGCA-3´
	Reverse	5´-TGCCACCACCGCTATTAAAGTCTGCCCGGCGA-3´
R422S	Forward	5´-CTTCCAGTCACCAGCGCTCTGCTGTCCTCCACC-3´
	Reverse	5´-GGTGGAGGACAGCAGAGCGCTGGTGACTGGAAG-3´
R407S	Forward	5´-GTGGTGGTGGTGGCAGCGGAGGATTTCCCAG-3´
	Reverse	5´-CTGGGAAATCCTCCGCTGCCACCACCACCAC-3´
R383S	Forward	5´-CGGTGGTGGCAATGGTAGTGGAGGCCGAGGG-3´
	Reverse	5´-CCCTCGGCCTCCACTACCATTGCCACCACCG-3´
R386S	Forward	5´-TGGTAGTGGAGGCAGCGGGCGAGGAGGACC-3´
	Reverse	5´-GGTCCTCCTCGCCCGCTGCCTCCACTACCA-3´
R388S	Forward	5´-TGGAGGCAGCGGGAGCGGAGGACCCATGGG-3´
	Reverse	5´-CCCATGGGTCCTCCGCTCCCGCTGCCTCCA-3´

### Statistical analyses

At least *n* = 3 biological experiments were performed for every statistical analysis using Microsoft Excel 2013; this includes having independent HEK293T cultures for each biological experiment. A Student's *t* test at 95% confidence was used for the comparison of two groups. Statistical analysis performed for fractionation experiments comparing the protein R.I. values uses a repeated-measures ANOVA and is estimated with a linear mixed model, which takes into account the experimental variance of replicates, and the dependence between the fractions of a same experiment the data were modeled with a heterogeneous first-order autoregressive structure. Each statistical analysis and the number of biological experiments are indicated in the figure legends. All statistical analyses considered *p* < 0.05 to be significant.

## Data availability

All data presented here are contained within the article.
