# Supplementary material for: FUS contributes to mTOR-dependent inhibition of translation
Source: J Biol Chem. 2021 Jan 13;295(52):18459–73. doi: 10.1074/jbc.RA120.013801 (PMC7939483; doi:10.1074/jbc.RA120.013801)
Supplement: Supplementary file 1 [file mmc1.pdf]

SUPPORTING INFORMATION

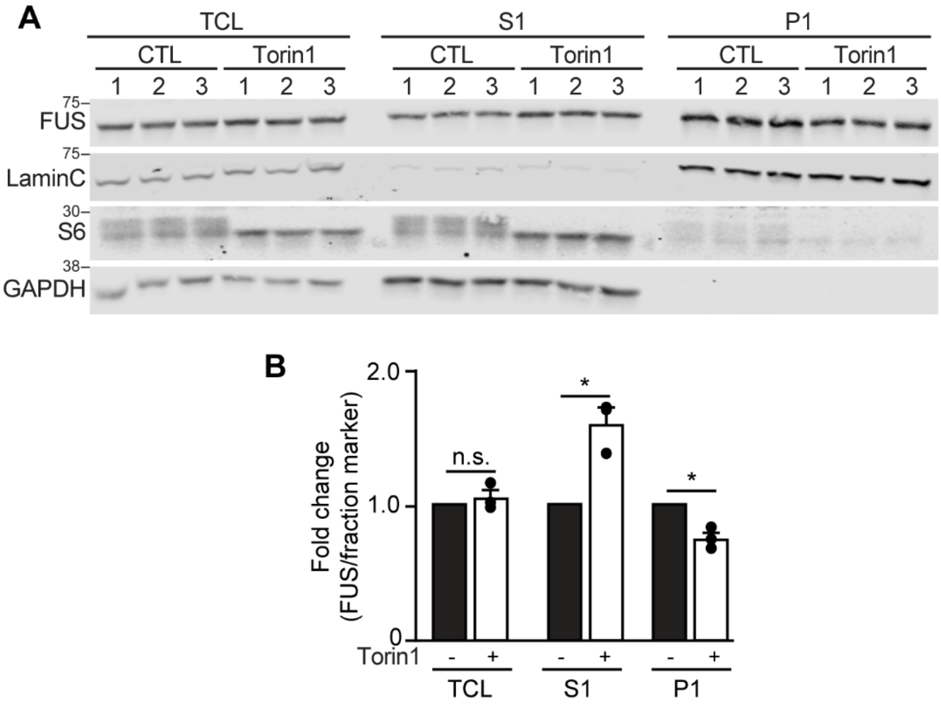

Figure S1. FUS localization in response to Torin1 treatment. Subcellular fractionation of HEK293T cells treated with DMSO (CTL) or Torin1 (250nM, 2 hrs). (*A*) Total cell lysate (TCL), soluble fraction (S1) and nuclear pellet (P1) were isolated and western blots were blotted with antibodies against FUS, S6, LaminC, and GAPDH. For each fraction, 2.0% of TCL and S1 and 0.3% of P1 were loaded. (*B*) Quantification of FUS in each fraction normalized to the corresponding fraction markers: LaminC (for P1) and GAPDH (for S1). Statistical analysis was performed using Student's *t*-test from n=3 biological replicates (n.s. = non-significant $p > 0.05$, $*p < 0.05$), error bars represent ± SEM.

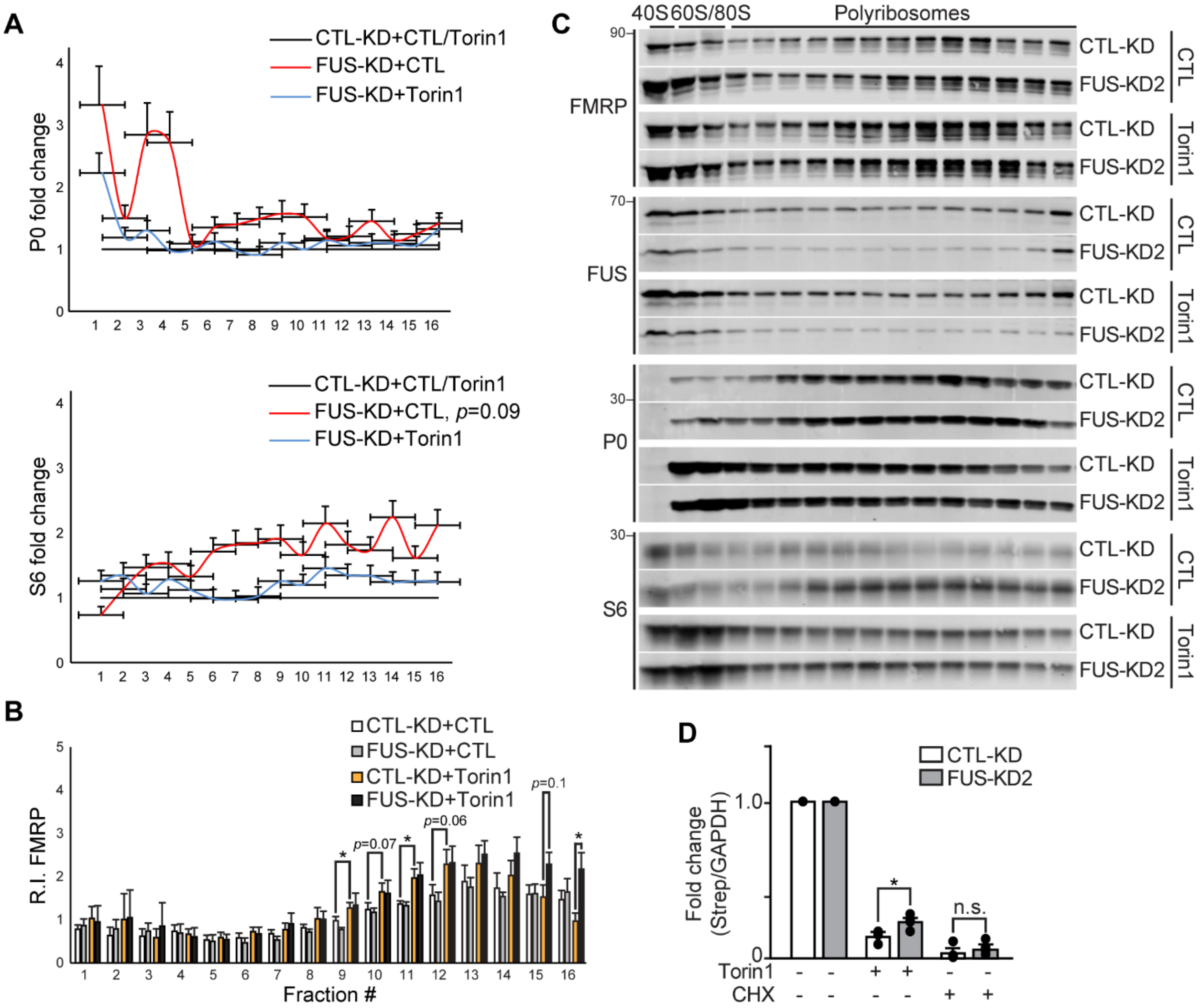

Figure S-2. FUS mediates Torin1-dependent translation inhibition. HEK293T cells were infected with shRNAs against a non-targeted shRNA (CTL-KD) or FUS (FUS-KD or FUS-KD2) and treated with DMSO (CTL) or Torin1 (250nM, 2hrs). (*A*) Quantification of the relative protein intensity (R.I.) for P0 (*top panel*) and S6 (*bottom panel*) are expressed as fold change relative to CTL-KD treated with DMSO (CTL) or Torin1. Statistical analysis uses a repeated measures ANOVA for n=4 biological replicates (S6 for FUS-KD+DMSO trends towards significance with p=0.09 relative to CTL-KD+CTL). (*B*). Quantification of the R.I for FMRP analyzed using a Student's *t*-test (*p < 0.05). (*C*) Western blots of proteins isolated from S1 sucrose gradient fractions of HEK293T cells infected with CTL-KD and FUS-KD2 were blotted with antibodies against FUS, FMRP and ribosomal proteins S6 and P0. Data shown are representative of n=3 biological replicates. (*D*) HEK293T cells infected with CTL-KD or FUS-KD2 were metabolically labeled with Click-iT® L-azidohomoalanine (AHA) to assess nascent protein synthesis. Shown is the quantification of AHA-labelled nascent proteins (Strep) relative to CTL-KD (DMSO), proteins were normalized to GAPDH. Statistical analysis of (*D*) was performed using a Student's *t*-test from n=4 biological replicates (n.s. = non-significant p > 0.05, *p < 0.05), *error bars* represent ± SEM.

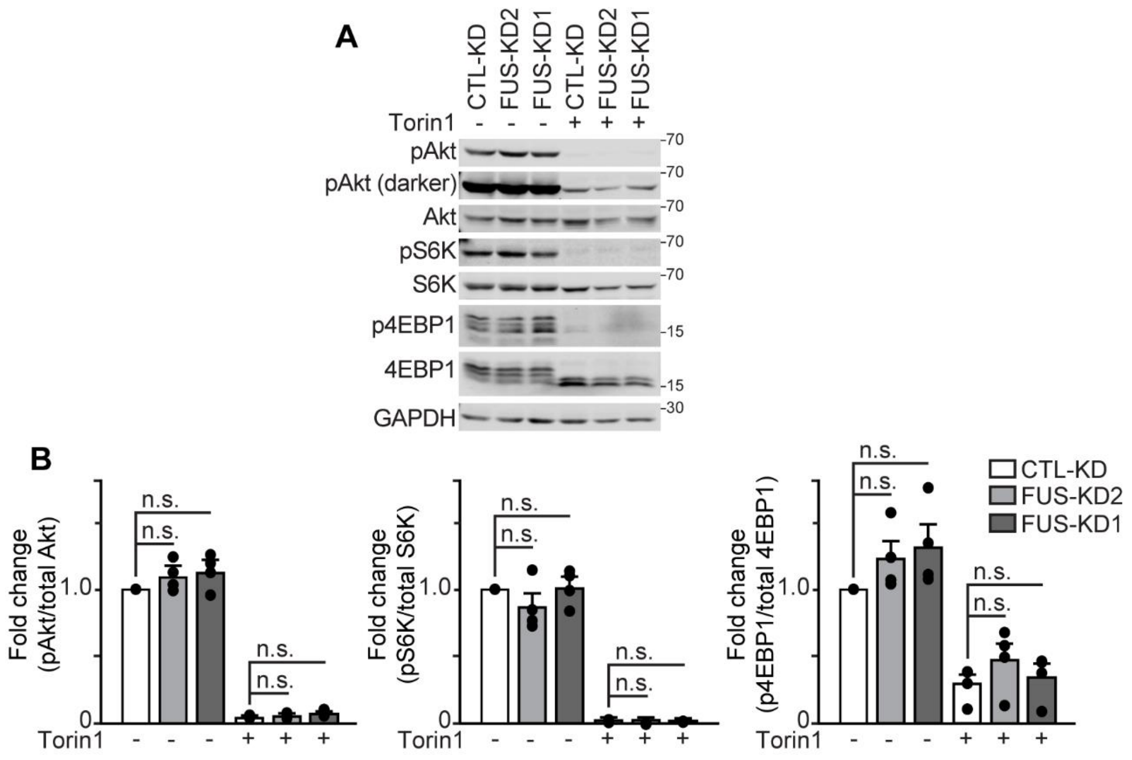

Figure S-3. Depletion of FUS from cells does not affect the down-stream targets of mTOR. HEK293T cells were infected with shRNAs against with a non-targeted shRNA (CTL-KD) or FUS (FUS-KD1 or FUS-KD2) and treated with DMSO or Torin1 (250nM, 2hrs). (*A*) Western blots of total cell lysates were blotted with antibodies against pAkt, Akt, pS6K, S6K, p4EBP1, 4EBP and GAPDH. (*B*) Quantification of the indicated proteins, which were normalized to GAPDH and then expressed as a ratio of phosphorylated /total proteins. The fold change of each protein is relative to DMSO. Statistical analysis was performed using a Student's *t*-test from n=4 biological replicates (n.s. = non-significant $p > 0.05$), *error bars* represent ± SEM.

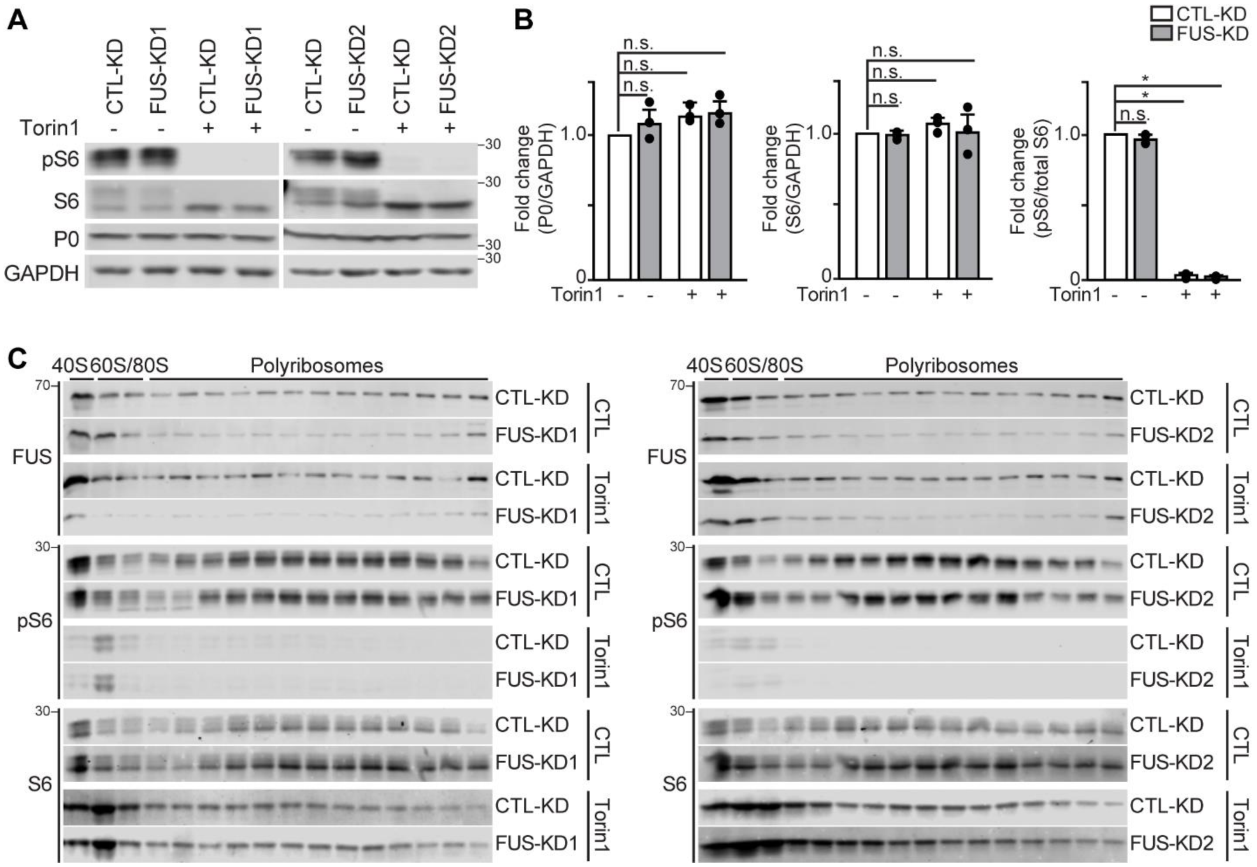

Figure S-4. Depletion of FUS from cells does not affect ribosomal markers. HEK293T cells were infected with shRNAs against a non-targeted shRNA (CTL-KD) or FUS (FUS-KD1 or KD2) and treated with DMSO (CTL) or Torin1 (250nM, 2hrs). (*A*) Western blots of S1 fraction inputs were blotted with antibodies against pS6, S6, P0 and GAPDH. (*B*) Quantification of P0 (*left panel*) and S6 (*middle panel*) and phosphorylated-S6 (ser 240/244)/total S6 from CTL-KD and FUS-KD S1 inputs, proteins were normalized to GAPDH. Statistical analysis was performed using a Student's *t*-test from n=3 biological replicates (n.s. = non-significant $p > 0.05$, $*p < 0.05$), *error bars* represent ± SEM. (*C*) Western blots of proteins isolated from S1 sucrose gradient fractions from HEK293T cell infected with CTL-KD, FUS-KD1 (*left panel*) or FUS-KD2 (*right panel*) were blotted with antibodies against FUS, phosphorylated and total ribosomal protein S6.

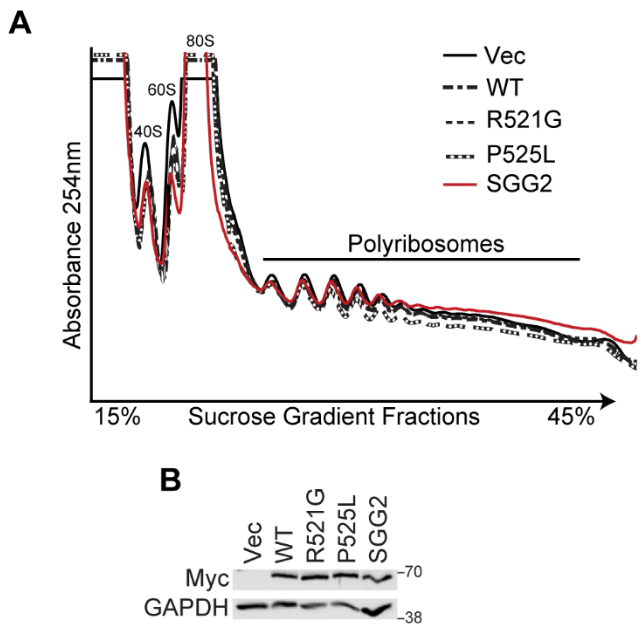

Figure S-5. ALS-FUS mutants repress translation in an RNA-dependent manner. HEK293T cells were transfected with empty vector pcDNA4b (Vec), or Myc-tagged FUS constructs: wild-type FUS (WT), ALS-FUS mutations (R521G and P525L) and FUS (SGG2), which has reduced binding affinity for RNA, for 48 hours before S1 extracts were subjected to polyribosome isolation by sucrose gradient centrifugation. (*A*) Absorbance (254 nm) trace of total RNA distribution. (*B*) Western blot of proteins from total cell lysates blotted with antibodies against Myc and GAPDH.
